# Hemostatic resuscitation for acute traumatic coagulopathy

**DOI:** 10.1186/1757-7241-19-2

**Published:** 2011-01-10

**Authors:** Thomas M Scalea

**Affiliations:** 1 Surgical Critical Care and Emergency Medicine, R Adams Cowley Shock Trauma Center, University of Maryland, Baltimore, USA

## Abstract

Trauma resuscitation paradigms have changed considerably over the last twenty years. Originally, the goal was to normalize a blood pressure as quickly as possible. Large volume crystalloid resuscitation was used to accomplish this. Standard therapy was that any patient with suspected bleeding received a two liter crystalloid bolus as initial therapy. It was often repeated and blood transfusion therapy was used relatively late. Fresh frozen plasma and platelets were also used relatively late, often after patients had received ten units of red cells. Dilutional anemia was relatively common. Patients with large volume blood loss often died from what was termed, "the bloody vicious cycle," of hypothermia, acidosis and coagulopathy.

## Commentary

Trauma resuscitation paradigms have changed considerably over the last twenty years. Originally, the goal was to normalize a blood pressure as quickly as possible. Large volume crystalloid resuscitation was used to accomplish this. Standard therapy was that any patient with suspected bleeding received a two liter crystalloid bolus as initial therapy. It was often repeated and blood transfusion therapy was used relatively late. Fresh frozen plasma and platelets were also used relatively late, often after patients had received ten units of red cells. Dilutional anemia was relatively common. Patients with large volume blood loss often died from what was termed, "the bloody vicious cycle," of hypothermia, acidosis and coagulopathy. Some of this was almost certainly iatrogenic from our resuscitation strategies [1].

The epidemic of urban violence that plagued large American cities in the late 1980's and early 1990's focused trauma surgeons on rethinking how we cared for serious injury. Damage control techniques were described and were incorporated into clinical practice [2]. In addition, use of deliberate hypotension was proven to be an effective strategy in the two randomized prospective trails that have been conducted in humans [3,4]. This is now widely practiced in many trauma centers, particularly in patients under the age of sixty-five years who do not have concomitant brain or spinal cord injuries.

Hemostatic resuscitation has also recently become a popular form of transfusion therapy. The concept of giving plasma and platelets early along with red cells in an attempt to closely approximate whole blood makes a lot of sense. In fact, when we reviewed blood usage at the Shock Trauma Center in the year 2000, massively transfused patients ultimately received a unit of plasma for every unit of blood that was transfused [5]. It made a lot of sense that giving FFP earlier would be beneficial. Clinical practice evolved even though there was a paucity of data. The military adopted hemostatic resuscitation. Data from the war certainly supports its use [6]. Civilian trauma centers followed. The new resuscitative paradigm has become to allow a systolic blood pressure to be around 80 mmHg, to limit crystalloid resuscitation, and use blood, FFP and platelets in a one to one to one ratio.

There are a number of recent civilian studies that comment on the efficacy of hemostatic resuscitation. Some have been done at single institutions [7,8]. Holcomb et al, however, reported a multicenter trial in sixteen U.S. level one trauma centers [9]. Patients who received high FFP and high platelet transfusion therapy had a significantly increased survival. In addition, those who died were significantly less likely to die from truncal hemorrhage and more likely to die from concomitant brain injury. That contrasts with our large single institution series that showed no difference in survival when a high FFP and red cell ratio was used [10]. The current paper [11] supports our work as it showed no difference in survival. How then can we reconcile these differences?

There are huge differences in trauma systems, in prehospital resuscitation strategies and transportation strategies. There can be little question that care provided in the prehospital phase has impact on long term outcome. Strategies such as "stay and play" vs. "load and go" may have some influence on survival. Some prehospital systems utilize physicians at the scene and en route to the trauma center. It is unclear whether this changes outcome but it almost certainly changes the process of care in the prehospital arena. Resuscitation strategies vary from institution to institution and from practitioner to practitioner. All of these may affect outcome. Yet, none of these are analyzed in any of the manuscripts published so far. These differences almost certainly persist after trauma center admission. Death after trauma can be from so many factors. Is it possible one change can affect outcome so markedly? It is hard to know the answer.

In the current manuscript, the authors changed their transfusion therapy and they then surveyed the time periods before and after these changes were made. 2004 was excluded as hemostatic resuscitation was introduced that year. They used an elegant statistical analysis to demonstrate that mortality did not change. A number of issues must be kept in mind when reading this manuscript. There is no description of cause of death. In fact, the same phenomenon may have occurred as described by Holcomb et al. Perhaps the cause of death changed and patients in the later group died from brain injury as opposed to exsanguination, or the affects of hemorrhage. In addition, no hemodynamic data is presented. While Injury Severity Score (ISS) is a good measure of anatomic injury, it is not a measure of the patient's physiology. Patients with multiple bony injury had the same ISS as did patients with severe torso injury. Yet, the rate of bleeding may be different. In fact, there was a statistically significant difference in the transfusions given in the first six hours when the early group was compared to the hemostatic resuscitation period.

Patients in the hemostatic resuscitation period got 20 units of blood in the first six hours after admission as opposed to 12 units during the earlier time period before changes were made. As the indication for transfusion did not apparently change, one might conclude that those patients were bleeding more quickly, and in fact were physiologically less stable. In addition, it would appear to me that virtually all of the blood was given early. Total transfusions were the same as the transfusions in the first six hours in the hemostatic resuscitation time period, and went up only minimally in the first time period. This would be different than transfusion practices in many trauma centers. The vast majority of patients presented in this series have blunt trauma. Many of them will have bled from bony injuries. This can certainly be impressive but it is different from true exsanguination from a grade V liver injury or significant intrathoracic injury. A better analysis might be to look at units of blood per hour as opposed to units of blood over a particular period of time.

There is little question that hemostatic resuscitation has changed the way that many trauma centers practice. While I have some concerns about this manuscript [11], this is clearly important work and is one of the few series that fails to demonstrate survival advantage from this new type of transfusion therapy. In fact, the authors are exactly correct when they conclude "prospective studies addressing the effect of various means of hemostatic controlled resuscitation in trauma patients presenting bleeding requiring transfusions are desperately needed." It is not until a true prospective randomized trail is undertaken, that we will really begin to start to answer the question of how much FFP is wise and when it ought to be utilized.

## Competing interests

The authors declare that they have no competing interests.
